# Gilteritinib and the risk of intracranial hemorrhage: a case series of a possible, under-reported side effect

**DOI:** 10.1007/s00277-023-05392-2

**Published:** 2023-08-22

**Authors:** Salvatore Perrone, Stefano Imperatore, Giuseppe Sucato, Ermanno Notarianni, Andrea Corbingi, Costanza Andriola, Mariasanta Napolitano, Alessandro Pulsoni, Matteo Molica

**Affiliations:** 1 Department of Hematology, S.M. Goretti Hospital, Polo Universitario Pontino, “Sapienza,” Via A. Canova, 04100 Latina, Italy; 2 Department of Health Promotion, Mother and Child Care, Internal Medicine and Medical Specialties (PROMISE), University of Palermo, Palermo, Italy; 3 Diagnostic and Interventional Unit, “Santa Maria Goretti” Hospital, Via Antonio Canova, Latina, Italy; 4 Department of Hematology-Oncology, Azienda Ospedaliera Pugliese-Ciaccio, Catanzaro, Italy

**Keywords:** Acute myeloid leukemia, Gilteritinib, FLT3, Intracranial hemorrhage (ICH), Subdural hemorrhage

## Abstract

Gilteritinib is currently approved for patients with relapsed/refractory AML with FLT3 mutations, based on the positive results of the pivotal ADMIRAL study. In ADMIRAL trial, no increased risk of bleeding was reported, but in the previous dose finding study, a single event of intracranial hemorrhage (ICH) was registered after exposure to subtherapeutic doses of gilteritinib. Here, we report the first case series on five ICHs diagnosed in patients with FLT3-mutated AML, occurred within the first month of exposure to gilteritinib. Our cohort included 24 patients treated in three Italian centers. Most of these ICH cases were non-severe and self-limiting, while one was fatal. This link with ICHs remains in any case uncertain for the presence of active AML. We further reported that an analysis of the post-marketing surveillance data (EudraVigilance) retrieved other 11 cases of ICHs present in the database after gilteritinib treatment. A causality assessment was performed according to the Dx3 method to evaluate the possibility that ICHs might be an actual side effect of gilteritinib. In conclusion, further research is needed to elucidate the potential role of gilteritinib in the pathogenesis of ICHs.

**Supplementary Information:**

The online version contains supplementary material available at 10.1007/s00277-023-05392-2.

## Introduction

FMS-related tyrosine kinase 3 (FLT3) mutations are detected in approximately 25–30% of acute myeloid leukemia (AML) patients, thus representing one of the most frequent mutations in AML [1]. Binding of FLT3 ligand (FLT3L) to FLT3 activates the phosphatidylinositol 3-kinase (PI3K) and RAS pathways, producing increased cell proliferation and inhibiting apoptosis [2]. Gilteritinib is currently the only FLT3 inhibitor approved for patients with relapsed or refractory (R/R) AML [3], based on the positive results of the pivotal ADMIRAL study: the median overall survival (OS) was significantly longer in patients treated with gilteritinib than among patients receiving any type of chemotherapy (9.3 vs. 5.6 months) [4]. The most frequent adverse events (AE) reported after gilteritinib administration were febrile neutropenia (45.9%), anemia (40.7%), and thrombocytopenia (22.8%); there is no report of an increased risk of bleeding or of intracranial bleeding episodes [4]. Nevertheless, in the previous phase I/II trial, which consisted of a dose finding of gilteritinib, a single event of intracranial hemorrhage (ICH) was registered after exposure to a subtherapeutic dose of the drug (20 mg) [5]. A new interim analysis from a post-marketing surveillance program for gilteritinib was recently published. In this analysis, conducted among 107 patients, 77.6% of patients presented an AE, but only 9.3% were hemorrhagic complications. Bleeding events of grade ≥3 were reported in 7% of patients experiencing an AE [6], and an Italian series reported 3 cases of gastrointestinal bleeding [7]. We describe the clinical history of five patients from three Italian institutions treated with gilteritinib for R/R FLT3-mutated AML, who developed ICH soon after exposure to the drug (some characteristics of these patients are summarized in Table 1). Patients provided informed consent to this retrospective analysis. The Ethics Committee of the University Hospital of Palermo first approved this retrospective study conducted retrospectively on patients treated with gilteritinib from May 2020 to May 2023, in 3 Italian hematology units (Catanzaro, Latina, Palermo). We also conducted a pharmacovigilance analysis using data reported on the website (www.adrreports.eu) of suspected adverse drug reactions from the European pharmacovigilance database (EudraVigilance, EV), accessed before June 6, 2023.

**Table 1:** Characteristics of patients who developed ICH

	Age	Sex	Genetics	1st line therapy	2nd line th.	Day of ICH	PLTs ×10^9^/L	CCT	Site	Outcome
#1	51	M	NPM1+ FLT3-TKD	7+3+mido		10	171	Normal	Subdural	Recover
#2	67	M	Relapse FLT3-TKD	5+2	Aza/ven	19	55	Normal	Subdural	Recover
#3	67	M	IDH1, Rel FLT3-ITD	Deci/ven		40	31	Normal	Parenchymal	Death
#4	37	F	Rel FLT3-ITD low	CPX-351	Allo-SCT	14	46	Normal	Parenchymal	Recover
#5	78	F	NPM1+ FLT3-ITD	5-Aza		12	31	Normal	Subdural	Recover

This table summarizes some characteristics of the five patients: age (years) at ICH; gender; molecular assets of FLT3 and other relevant genes; the first- and second-line therapy received; the days of manifestation of intracranial hemorrhage (ICH) from starting of gilteritinib; the number of platelets; the day of ICH; the conventional coagulation tests (CCT) including prothrombin time (PT), partial thromboplastin time (PTT), and fibrinogen; the site where ICH occurred; and the outcome of ICH

## Patient 1

**Fig. 1:**
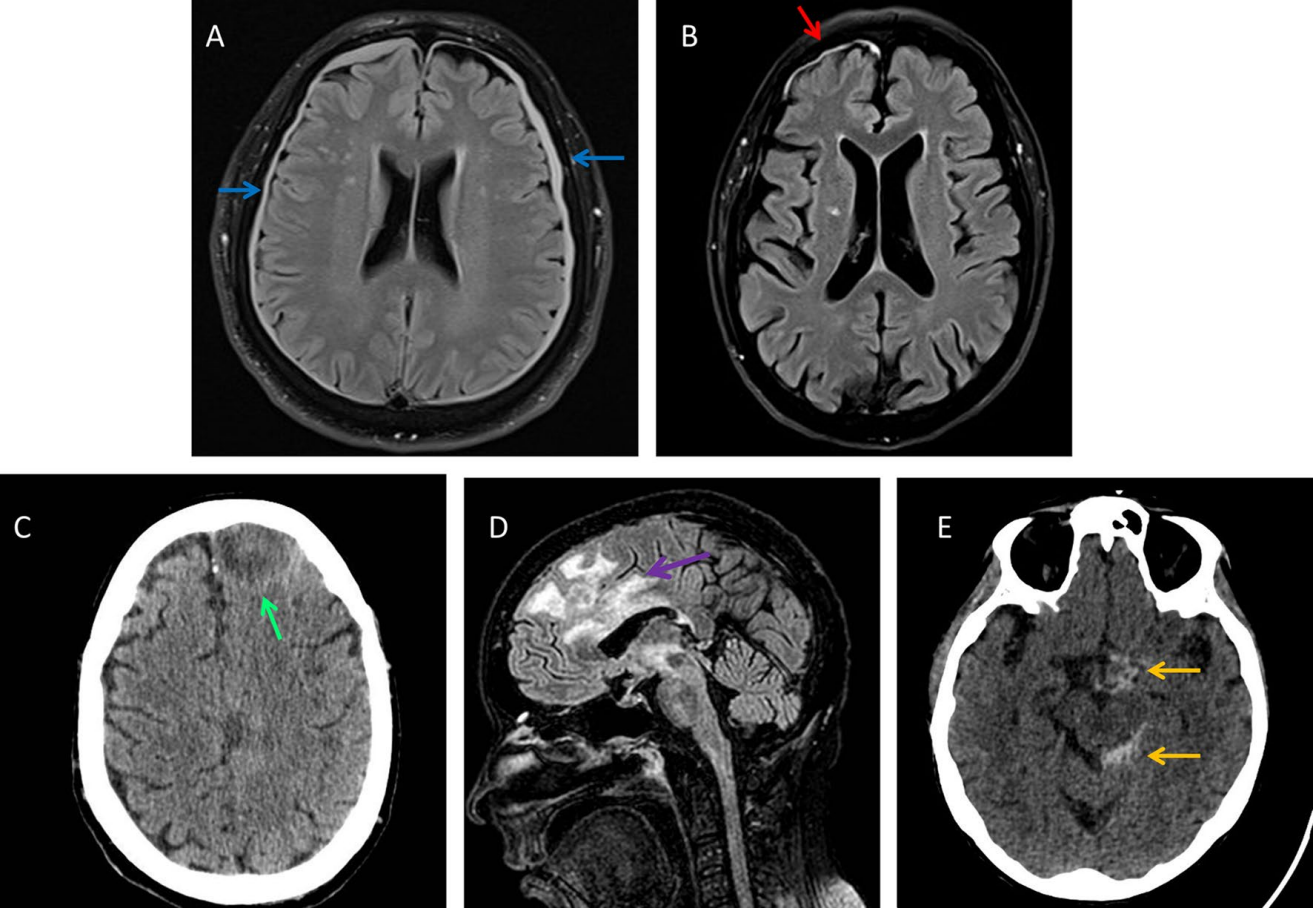
**A** Brain MRI documents bilateral subdural hematoma (blue arrows) in the frontal and parietal lobe (4–5 mm maximum thickness) with a thin and diffuse dural hyperintense FLAIR signal. **B** Frontal right subdural effusion (9 mm thick, red arrow) shows hyperintense FLAIR signal and heterogeneous but largely hyperintense T1 signal (not shown); meninges in that site show intense contrast enhancement (not shown). These findings could be referred to a chronic subdural hematoma with signs of more recent bleeding. **C** CT scan shows a heterogeneous and blurred hypodense lesion with a rim-like hyperdense tissue (green arrow) in the left paramedian frontal lobe (about 3.8 × 2.8 cm). **D** MRI of the sagittal plane documenting an expansive lesion (35 × 20 mm maximum) mainly in thalamic region with irregular margins (purple arrow), hyperintense FLAIR signal; the lesion extends caudally up to the homolateral midbrain and determines edematous-malacic effects up to the pons (more to the right), significant compressive phenomena on the lateral ventricle and initial contralateral midline shift (not shown). The picture is to be related to likely hemorrhagic-expansive lesion. **E** CT scan shows two hyperdense zones of subdural hemorrhage contouring the left perimesencephalic cisterns and the suprasellar cistern (orange arrows)

In March 2022, a 51-year-old male patient was diagnosed with AML (FAB M5). Blasts were NPM1-positive, FLT3 (D835+) mutated, and the karyotype was abnormal (47, XY; +8; iso21). Therefore, he was enrolled in GIMEMA protocol AML1919, which comprises an induction chemotherapy (CHT) regimen “7+3” + midostaurin and consolidation with intermediate doses of ARA-C (IDAC) + midostaurin. A morphologic complete response (CR) was achieved after induction CHT, but due to measurable residual disease (MRD) positivity (NPM1+) after consolidation, an Allo-SCT had to be considered. Unfortunately, at that time, no sibling donor was available, which required starting the search for a matched unrelated donor (MUD). In the meantime, the patient received a second consolidation course with IDAC+midostaurin in May. In July, Allo-SCT screening was started. Pending this, the patient contracted a SARS-CoV2 infection. This resolved in September 2022, when a bone marrow aspiration documented a relapsed disease (FLT3 TKD+). In early October, salvage treatment was started with gilteritinib (120 mg/day). A complete blood cell count (CBC) showed hemoglobin (Hb)= 11.3 g/dL, white blood cell count (WBC) 12.25× 10^9^/L, neutrophil count (N) 10.13× 10^9^/L, and PLT (118× 10^9^/L), and conventional coagulation tests (CCT including prothrombin time (PT), partial thromboplastin time (PTT), and fibrinogen) were normal (PT/INR= 0.91, PTT=25 s, fibrinogen=617 mg/dL). Ten days after the start of gilteritinib, a brain MRI was performed for a persistent headache, which showed a bilateral subdural hematoma (Fig. 1A), not requiring neurosurgical draining. Treatment with gilteritinib was continued. One month later, the subdural hematoma improved at neuroimaging, and the bone marrow examination showed a CR. The patient successfully received an Allo-SCT in the absence of further bleeding complications.

## Patient 2

A 67-year-old man was diagnosed in August 2018 with (FAB M4)-AML, with no valuable karyotype and no molecular risk factors (NPM1-; FLT3 (ITD or TKD) -). Due to his age and comorbidities (hypertension and diabetes), he received induction with ARA-C and daunorubicin at reduced doses (5+2 protocol), which produced no response. A second-line therapy was then attempted with azacitidine + venetoclax for six cycles. A CR was achieved after the fourth cycle. The patient, however, refused Allo-SCT and then continued for another 10 cycles of AZA+Ven, until December 2019. In May 2020, a first relapse was documented at bone marrow aspiration, with 70% of blasts carrying the mutation in FLT3-TKD (D835). On July 1, the patient started gilteritinib (120 mg/day), with the following CBC and CCT results: Hb= 7.9 g/dL, WBC =8.53× 10^9^/L, N= 0.41× 10^9^/L, and PLTs= 30× 10^9^/L. Nineteen days after treatment initiation, he presented to the ER for seizures and syncope. Gilteritinib was suspended, the CBC showed Hb 10.8g/dL, WBC 2.31×10^9^/L, N 0.5×10^9^/L, and PLTs 55×10^9^/L (PT/INR 1.2, PTT 27 s, fibrinogen 856 mg/dL), and an MRI documented a 9 mm subdural hematoma of the right frontal lobe (Fig. 1B). This bleeding episode was managed conservatively with an antiepileptic drug (levetiracetam). The patient improved, and after only 3 weeks (on July 24), gilteritinib was re-started in the absence of further bleeding episodes. Gilteritinib was administered only until D+28, when it was definitely discontinued due to disease progression. The patient was then recommended for palliative care.

## Patient 3

In August 2021, a 67-year-old man was diagnosed with AML with the following biological characteristics: normal karyotype, FLT3 unmutated, NPM1 unmutated, and IDH1 mutated. At the time of AML diagnosis, he presented a dilated cardiomyopathy with an ejection fraction of 37%, which led to the decision to not administer intensive chemotherapy. The patient received a combined treatment of decitabine at the standard dose and venetoclax, administered continuously at the dosage of 100 mg/day, associated with prophylactic antifungal treatment (posaconazole). After six cycles of treatment, the patient presented with hyperleukocytosis and approximately 60% blasts. Retesting was performed, which detected a FLT3-ITD mutation at a high allelic ratio (AR>0.5). Treatment with gilteritinib at the standard dose of 120 mg/day was immediately started. At that time, CBC showed Hb = 8.7 g/dL, WBC= 48.2×10^9^/L, and PLTs= 56×10^9^/L with normal CCT. After 40 days of treatment, the patient showed a sudden deviation of the lip rhyme, associated with right hemiplegia. A cranial CT scan documented a hemorrhagic effusion localized in the left frontal area with a maximum size of about 3.6 × 2.8 cm (Fig. 1C). The CBC and CCT showed Hb 9.6 g/dL, WBC 12.8×10^9^/L, N 0.43×10^9^/L, PLTs 31×10^9^/L, PT= 12.9 s, PTT= 28.3 s, and fibrinogen=451 mg/dL. Gilteritinib was discontinued; the patient was hospitalized and, after 1 month, died from further neurological complications.

## Patient 4

In January 2022, a 37-year-old woman was diagnosed with AML classified as high risk according to ELN2017 (no recurrent mutations, complex karyotype). The patient had a history of breast cancer and was treated with chemotherapy and radiotherapy at the age of 34. She was treated with induction and two consolidation cycles of CPX-351, achieving a CR. Thereafter, she received a bone marrow Allo-SCT from a MUD. Forty-seven days after transplant, the patient showed an AML relapse, almost entirely losing the chimerism of the donor. She was then retested for molecular mutations, and a FLT3-ITD mutation at a low allelic ratio was detected. The patient was immediately treated with gilteritinib at the standard dose of 120 mg/day. After only 2 weeks of gilteritinib treatment, the patient had an epileptic crisis requiring hospitalization. MRI documented a 2 × 2 cm hematoma in the left parietal lobe (Fig. 1D). CBC showed that Hb 11.2 g/dL, WBC 3.88×10^9^/L, N 0.17×10^9^/L, and PLTs 46×10^9^/L. CCT were within the normal range (PT=11.2 s, PTT=28 s, fibrinogen= 310 mg/dL). After 2 weeks, the patient died due to septic shock.

## Patient 5

In April 2023, a 78-year-old woman was diagnosed with relapsed NPM1-positive, FLT3-ITD-mutated AML after 38 cycles of azacytidine. CBC showed that Hb 7.1 g/dL, WBC 68.6×10^9^/L, N 11.8×10^9^/L, PLTs 19×10^9^/L, and blasts 68%. Two months before her relapse, she was also diagnosed with breast cancer, but surgery had to be postponed due to the patient’s generally poor conditions and worsening blood counts. Gilteritinib was tentatively administered. After 12 days, however, the patient became progressively disoriented and confused, particularly overnight. This resulted in a fall and a close facial trauma. Head CT showed subarachnoid hemorrhage involving the left frontotemporal region, extending to the pontine cistern and left peri-mesencephalic cisterns, and to the ipsilateral suprasellar cistern (Fig. 1E). On that day, CBC showed that Hb 8.7 g/dL, WBC 0.62×10^9^/L, N 0.12×10^9^/L, and PLTs 31×10^9^/L. CCT were within the normal range (INR= 0.94, PTT=21 s, fibrinogen=522 mg/dL). The 1-month marrow showed CRi, but several infections, including pneumonia, complicated the clinical setting. The patient improved neurologically and was referred to palliative care after 2 months.

## Discussion

This study describes the largest casuistry of early intracranial bleedings occurring in five patients treated with gilteritinib up-to-date. In our 3 centers, a total of 24 AML patients were treated with gilteritinib from May 2020 to May 2023. ICH was observed in five of them (6/24, 20%). The most important limitation of the current report, apart from the small numbers, is represented by its retrospective nature.

For each case, a causality assessment was performed according to the Dx3 method [8]. This approach allows a qualitative assessment of the relationship between the use of a drug and AEs, by using a checklist to guide the analysis of the following three domains: the drug disposition, the pre-disposition of the patient (vulnerability), and the disposition of the patient–drug interaction (mutuality).

### Drug disposition

In a Japanese post-marketing surveillance study on patients receiving gilteritinib, hemorrhage was reported in 9.3% of them [6]. Given this, it is only possible to speculate about several potential mechanisms involved in these bleeding manifestations and their cerebral localization. The rate of ICH of about 20% observed in our study is higher than that reported in the literature for this event at diagnosis of AML [9–12].

Gilteritinib seems to have several, still poorly explored, mechanisms of action. The manner in which it could be related to ICH is unknown. Gilteritinib inhibits glutamine uptake and utilization by interfering with glutamine transporters (i.p.SLC38A1) [13]; reduced glutamine availability contributes to cell senescence and death. Glutathione protects from oxidant radicals which in turn interfere also with clot stability. Gilteritinib may therefore potentially lead to coagulation derangements by decreasing fibrin polymerization and impairing clot stability [14]; glutathione also contributes to regulating platelet homeostasis [15]; the circumstance that cerebral tissue metabolism is deeply oxygen dependent might explain the occurrence of bleeding mainly in the cerebral tissues, even if gilteritinib concentrations are commonly considered to be quite low in the cerebrospinal fluid [16, 17]. These hypotheses are supported by the occurrence of ICH in the presence of concomitant factors that contribute to impairing the CNS barrier function and increasing its permeability (previous radiotherapy, glioblastoma).

Gilteritinib may also directly affect platelet functions and indirectly megakaryocytes [18]. Twenty-two percent of patients treated with this drug developed thrombocytopenia [4], and gastrointestinal bleeding has been reported in animal models and in the AZA/gilteritinib trial [7, 19]. The drug may also exert a direct action on the brain vasculature. It is involved in the development of posterior reversible encephalopathy syndrome (PRES). PRES occurred in two patients (1%) treated with gilteritinib out of 319 patients in the integrated safety evaluation from the ADMIRAL trial. Events occurred on days 67 and 276 of therapy. PRES symptoms included seizures and altered mental status, which resolved after discontinuation of gilteritinib [20]. PRES may be related to endothelial cell dysfunction/injury, leading to blood-brain barrier leakage, with resultant cortical and subcortical vasogenic edema. PRES may be complicated by cerebral hemorrhage (in 15% of patients) [21]. Therefore, the high prevalence of ICH observed in our series may be related to the fact that we requested more neuroimaging to rule out a PRES syndrome that requires discontinuation of gilteritinib, but we incidentally found ICH. In conclusion, there is moderate evidence that there is a correlation between gilteritinib and ICHs.

### Patient vulnerability

None of the above reported patients had experienced ICH events before the exposure to gilteritinib. However, several confounding factors are present in this setting of r/r AML patients. ICH is quite frequent in patients with AML; several studies have reported an incidence of ICH ranging from 8 [9] to 6.1% [10], reduced to 4% of subjects when PLT transfusion is prophylactically administered [11]. Bleedings usually occur in the first 7 days after AML diagnosis [12]. Some risk factors for ICH, such as female gender and reduced fibrinogen plasma levels, have been identified [11]. Moreover, severe thrombocytopenia and hyperleukocytosis at diagnosis of acute leukemia are well-known risk factors for ICH [22, 23]. In our series, bleeding could be related to the low platelet count, secondary both to the disease or to the hematological toxicity of gilteritinib. Indeed, in our series, all patients had PLT>30 × 10^9^/L when bleeding occurred. Importantly, CCT, including fibrinogen levels, were within the normal range in all the patients here reported. Therefore, there is good evidence for the vulnerability of the patients to the reported events.

### Mutuality

The temporal development (in all our cases, the median interval from gilteritinib starting to ICH was 13 days) of the events indicates a plausible interaction between the patients’ disposition and the drug’s properties. Therefore, there is moderate evidence of drug–patient interaction.

## Data from the EudraVigilance database

By June 5, 2023, 950 individual cases had been identified in the EudraVigilance database for gilteritinib. Of these, we found in the subcategory of nervous system disorders that one fatal case of intracranial hemorrhage was reported and another 10 cases of cerebral hemorrhage (8 in the 65–85-year group and 1 in the 18–64-year group). The outcome resulted fatalities in 4 cases, no recovery in 2 cases, and recovery in 2 cases (Supplemental 1). These post-marketing reports underline the occurrence of similar AEs in other European datasets.

In conclusion, this is the first case series to report on five early ICHs in patients with AML FLT3-mutated, in the first month after the beginning of gilteritinib. Most of these cases were mild and self-limiting, while in one case, ICH resulted in death, but the concurrent presence of active AML prevents a clear link with the drug. However, further studies are needed to adequately define the incidence of bleeding complications after gilteritinib administration in a real-life setting and also to understand the mechanisms of this potentially life-threatening complication. The commonly adopted laboratory assays are currently unable to predict bleeding risk in this setting; further research is thus needed also for bleeding risk prediction [24]. In the meantime, we encourage early suspicion of ICH in patients on gilteritinib presenting with even mild symptoms (e.g., headache and seizures) and prompt ordering of neuroimaging exams.

### Supplementary information

ESM 1

## Data Availability

Not applicable.
