# Supplementary figures and images for: Gilteritinib and the risk of intracranial hemorrhage: a case series of a possible, under-reported side effect
Source: Ann Hematol. 2023 Aug 22;102(11):3025–30. doi: 10.1007/s00277-023-05392-2 (PMC10567884; doi:10.1007/s00277-023-05392-2)

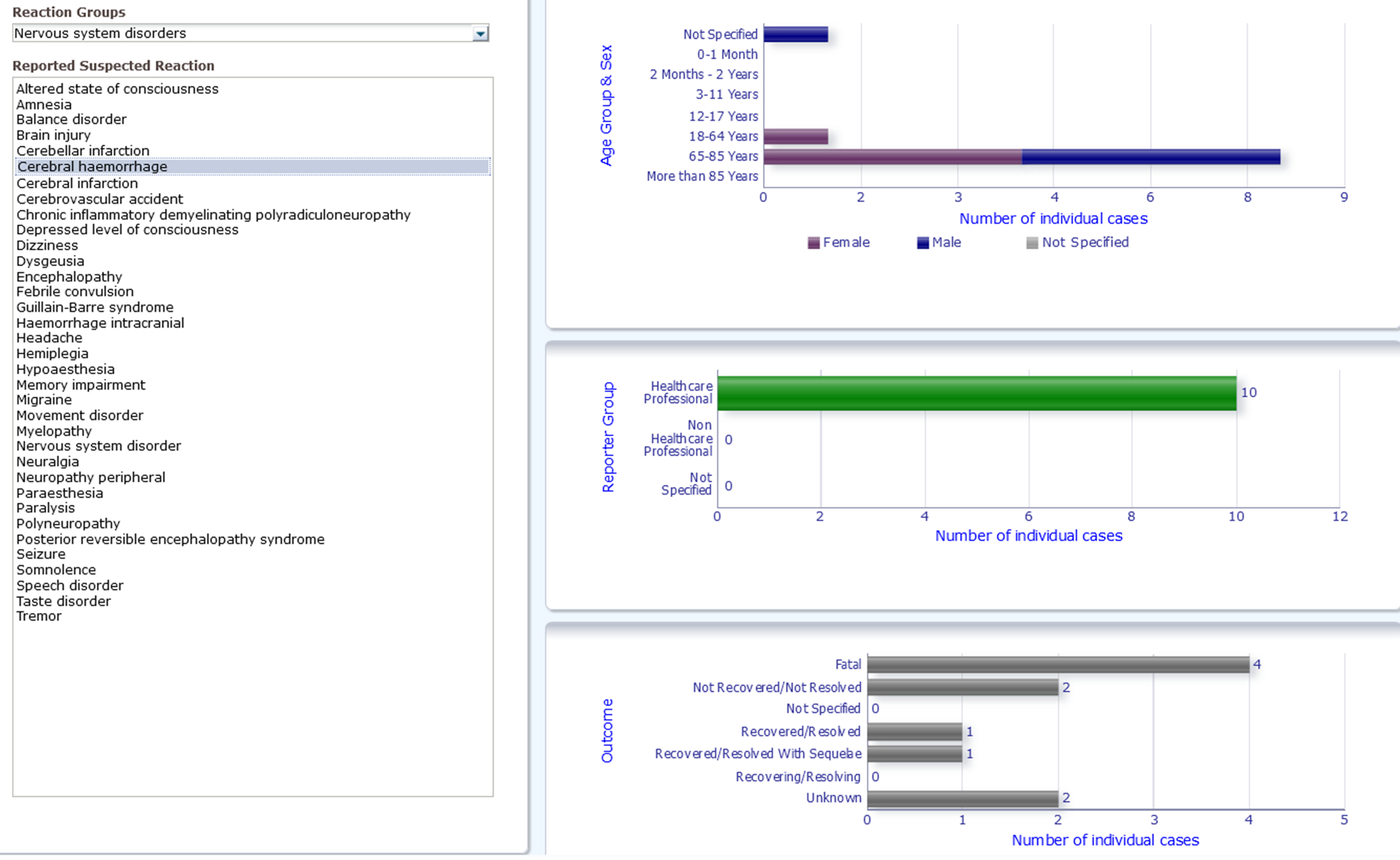

Supplement: Supplementary file 1 — ESM 1 [file 277_2023_5392_MOESM1_ESM.png]
